# Activation of the cGAS–STING signaling pathway in adenomyosis patients

**DOI:** 10.1002/iid3.452

**Published:** 2021-05-19

**Authors:** Yun Lin, Luying Wang, Mingzhu Ye, Ke‐nan Yu, Xin Sun, Min Xue, Xinliang Deng

**Affiliations:** ^1^ Department of Obstetrics and Gynecology The 3rd Xiangya Hospital of Central South University Changsha Hunan China

**Keywords:** adenomyosis, endometrium cGAS, STING

## Abstract

**Objective:**

Adenomyosis is characterized by the presence of endometrium or endometrium‐like glands and stroma within the myometrium. In this study, we aimed to investigate whether the cGAS–STING pathway was activated and correlated with clinical outcomes in adenomyosis patients.

**Materials and Methods:**

Twenty patients diagnosed with adenomyosis and 10 patients diagnosed with cervical intraepithelial neoplasia grade 3 (CIN‐3) but no adenomyosis were enrolled in this study. Specimens were collected during surgery from August 2017 to December 2017 at Third Xiangya Hospital. The messenger RNA (mRNA) and protein levels of key cGAS–STING pathway factors in uterine tissue were detected by real‐time reverse‐transcription polymerase chain reaction and immunohistochemistry, respectively. The correlations of gene expression and clinical outcomes, including dysmenorrhea and uterine volume, were analyzed.

**Results:**

The cGAS, STING, TANK‐binding kinase 1 (TBK‐1), interferon‐α (IFN‐α), IFN‐β, and tumor necrosis factor‐α (TNF‐α) mRNA and protein levels in the ectopic endometrial tissue from adenomyosis patients were significantly higher compared with that from the controls in endometrium (*p* < .05). cGAS and STING gene expression were correlated with TBK‐1, IFN‐β, and TNF‐α expression (*p* < .05). Importantly, TBK‐1 and TNF‐α expression were correlated with the clinical outcome of dysmenorrhea (*p* < .05).

**Conclusion:**

Our study reveals that the cGAS–STING pathway is activated in adenomyosis patients and its activation is subsequently correlated with clinical outcomes, which suggests that the cGAS–STING pathway may contribute to adenomyosis pathogenesis.

## Graphical abstract

Representative pelvic magnetic resonance imaging (MRI) from the control and adenomyosis patients. A. In a control patient (control), the MRI picture shows the normal uterine, with dimensions of a 45.6‐mm length and 43.1‐mm width crossing the whole uterus. B. In an adenomyosis patient (Aden.), the MRI scan shows typical focal adenomyosis, with a 40.6‐mm length and 56.9‐mm width of pathological foci located in the uterine junctional zone (indicated with a red star).

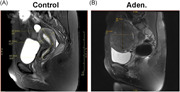

## 1 INTRODUCTION

Adenomyosis is a common gynecologic disease. Histological examination shows endometrial glands and stroma deep presented within the myometrium.^1^ Most women with adenomyosis are asymptomatic,^2^, ^3^ while others may suffer from a spectrum of symptoms including dysmenorrhea, dyspareunia, abnormal uterine bleeding, and infertility.^2^, ^3^ However, the pathophysiology of adenomyosis is still poorly understood.^4^, ^5^ Several theories have been proposed for the etiology and pathogenesis of adenomyosis, including down growth and invagination of the basalis endometrium into the myometrium^6^, ^7^ and local hyperestrogenism, which may contribute to adenomyotic development.^8^, ^9^, ^10^ One previous study showed that the local estrogen level was increased in adenomyosis patients, which induced hypertrophy and hyperplasia in the surrounding myometrium and overlying endometrium.^9^ In addition, an abnormal immune response might facilitate the pathological process of adenomyosis.^4^, ^11^

Recently, accumulating evidence has indicated that abnormal immune responses may also play an important role in adenomyotic development. Li et al.^12^ found that expression of the nuclear factor‐κB (NF‐κB) subunits p50 and p65 was significantly increased and NF‐κB DNA‐binding activity was significantly higher in adenomyosis patients than that in the controls. An in vitro experiment using uterine smooth muscle cells derived from myometrium biopsies of adenomyosis patients showed that the MAPK/ERK cell signaling pathway was activated.^13^ Furthermore, increase of interleukin‐8 (IL‐8),^14^ monocyte chemoattractant protein‐1 (MCP‐1),^14^ and IL‐10 expression in local tissue^15^ and significant elevation of IL‐37 and IL‐10 but decrease of IL‐17A expression in adenomyosis patient sera were detected.^16^ Increased IL‐1β, CRH, and UCN expression in adenomyotic nodules further supports the involvement of inflammation in adenomyosis pathogenesis.^17^

The cGAS–STING pathway is a recently identified novel innate immune recognition pathway. Initially, cytosolic DNA binds to cGAS, which promotes cGAS to undergo a conformational change that allows ATP and GTP to be synthetized into cyclic GMP‐AMP (cGAMP). cGAMP binds to and activates the ER membrane adaptor STING. Then, STING activates a kinase (either TANK‐binding kinase 1 [TBK‐1] or IκB kinase [IKK]) to trigger transcription of interferon and inflammatory cytokines, such as tumor necrosis factor (TNF), IL‐1β and IL‐6.^18^, ^19^, ^20^ The cGAS–STING pathway has been reported to play a crucial role in immune defense against various DNA viruses,^21^ certain retroviruses,^22^ and intracellular bacteria^23^ and to sense mitochondrial DNA (mtDNA) under cellular stress conditions.^24^ The pathway can even be activated by self‐DNA in autoimmune diseases.^25^

Histopathological examination of adenomyosis patient specimens in our clinic has revealed significant inflammatory cell infiltrations into the uterine tissue, especially in the ectopic endometrial glands, which suggested that an inflammatory immune response was involved in adenomyosis. We hypothesized that the cGAS–STING pathway might be activated in local tissue in adenomyosis patients and played an important role in adenomyotic development. Therefore, in the current study, we investigated cGAS–STING pathway activation in uterine adenomyotic lesion tissues by quantitative real‐time reverse‐transcription polymerase chain reaction (qRT‐PCR) and immunohistochemical staining and analyzed the correlation of key cGAS–STING pathway factor expressions and the clinical outcomes in adenomyosis patients.

## 2 MATERIALS AND METHODS

### 2.1 Patients and tissue collection

From August 2017 to December 2017, patients who underwent hysterectomy in our hospital with a histopathological diagnosis of adenomyosis were enrolled and served as the adenomyosis group (*n* = 20). The patients who underwent hysterectomy and were histopathologically identified as cervical intraepithelial neoplasia grade 3 (CIN‐3) without adenomyosis were set as the control group (*n* = 10). Exclusion criteria were patients with viral and bacterial infection, cancer and malignant disease, autoimmune disease, or any major operation within the previous 3 months. We chose the CIN‐III patients but adenomyosis negative as controls because: (1) For certain amounts of patients, especially for those older patients who would like to perform the hysterectomy when examined with CIN‐III. (2) we did magnetic resonance imaging (MRI) for CIN III patients to exclude uterine body disease. (3) There are no biases of how we select these control patients. If there is a coexisting disease, it will be excluded from the study subject. For adenomyosis group, the adenomyotic tissue samples were collected during hysterectomy, while for the controls their endometrial tissues were harvested. Each piece of tissue from every patient was divided into two parts. The first part was cut into 5‐mm cubes, chopped and stored in RNAlater for future RNA extraction, and the second part was fixed in 4% paraformaldehyde solution for subsequent immunohistochemistry (IHC) staining.

The clinical characteristics, including age, number of pregnancies, menstrual cycle length (is counted from the first day of one period to the first day of the next menstrual cycle), menstrual period length (the number of continuous days of bleeding within each of the menstrual cycles), dysmenorrhea (evaluated with a 10‐point pain scale), uterine volume and MRI scan pictures were recorded during clinic visits and hysterectomy surgery. This study was approved by the Review Board and Ethics Committee of the Third Xiangya Hospital of Central South University (No.2016‐S010). All patients signed a statement of consent to participate under the “ethics, consent, and permissions” heading and another informed consent form for the publication of the collected data.

### 2.2 RNA extraction and real‐time PCR

The qRT‐PCR procedure was described previously.^26^ Briefly, total RNA was isolated from uterine tissue by TRIzol reagent (Invitrogen). Complementary DNA (cDNA) was synthesized from 1 μg of RNA with the Maxima First‐Stand cDNA Synthesis Kit (Fermentas) according to the manufacturer's instructions. Real‐time PCR was performed with the 7500 Real‐Time PCR System (Applied Biosystems) and the 2 × Maxium SYBR Green/ROX qPCR Master Mix (Fermentas) according to the manufacturers' instructions. The primers used in this study were shown in Table 1. The relative transcript levels were calculated with the 2‐ΔΔCt method and normalized to glyceraldehyde 3‐phosphate dehydrogenase (GAPDH), which served as an internal standard control. Every gene from each patient was analyzed in triplicate, and the final data were calculated and presented as the mean ± *SEM*.

**Table 1:** Primers

Primers	Sequence
GAPDH forward	5ʹ‐ACAGCCTCAAGATCATCAGC‐3ʹ
GAPDH reverse	5ʹ‐GGTCATGAGTCCTTCCACGAT‐3ʹ
cGAS forward	5ʹ‐CTCCACGAAGCCAAGACCTC‐3ʹ
cGAS reverse	5ʹ‐GCGGCTGAGCTTCAACTTCT‐3ʹ
STING forward	5ʹ‐CCTGTTGCTGCTGTCCATCT‐3ʹ
STING reverse	5ʹ‐ATGTTCAGTGCCTGCGAGAG‐3ʹ
TBK‐1 forward	5ʹ‐GGAAGCGGCAGAGTTAGGTG‐3ʹ
TBK‐1 reverse	5ʹ‐TCGGATGAGTGCCTTCTTGA‐3ʹ
IFN‐α forward	5ʹ‐GCCATCTCTGTCCTCCATGA‐3ʹ
IFN‐α reverse	5ʹ‐GCTGGTAGAGTTCGGTGCAG‐3ʹ
IFN‐β forward	5ʹ‐GCCGCATTGACCATCTATGA‐3ʹ
IFN‐β reverse	5ʹ‐AGTCTCATTCCAGCCAGTGCT‐3ʹ
TNF‐α forward	5ʹ‐TGGAGAGTGAACCGACATGG‐3ʹ
TNF‐α reverse	5ʹ‐CTCTCAGCTCCACGCCATT‐3ʹ

Abbreviations: GAPDH, glyceraldehyde 3‐phosphate dehydrogenase; IFN‐α, interferon‐α; TBK‐1, TANK‐binding kinase 1; TNF‐α, tumor necrosis factor‐α.

### 2.3 IHC staining

The IHC staining procedures were described previously.^27^ Briefly, formalin‐fixed uterine tissue was serially sectioned at a 5‐µm thickness and then deparaffinized and rehydrated in a graded ethanol series and washed in Tris‐buffered saline (20 mmol/L of Tris–HCl and 150 mmol/L of NaCl [pH 7.6]). Antigen retrieval was performed by boiling the slides in sodium citrate buffer (10 mmol/L, pH 6.0) for 15 min. After blocking with 10% FBS buffer, primary antibodies, including anti‐cGAS (rabbit anti‐human C6orf150; Proteintech; cat #26416‐1‐AP), anti‐STING (rabbit anti‐human TMEM173; Proteintech; cat #19851‐1‐AP), anti‐TBK‐1 (rabbit anti‐human TBK‐1; Abcam; cat #ab40676), anti‐IFN‐α (rabbit anti‐human IFN‐α; Bioss; cat #bs‐1578R), anti‐IFN‐β (rabbit anti‐human IFN‐β; Bioss; cat #bs‐0787R), and anti‐TNF‐α (mouse anti‐human TNF‐α; Proteintech; cat #60291‐1‐Ig), were used to probe for endogenous proteins in the uterine tissue by incubation at 4°C overnight. After washing with PBS, the tissue slides were incubated with the secondary antibody. After additional washing, the slides were stained with 3,3ʹ‐diaminobenzidine. The staining was closely monitored, and the slides were immersed in distilled water to stop the reaction as soon as the color developed. Then, the sections were counterstained in hematoxylin for 20–40 s, washed with tap water, and mounted with 100% glycerol.

The immunohistochemistry staining images were captured under a microscope, and representative images were presented. For quantitative calculation of the immunohistochemistry staining, the integrated optical density (IOD) per high‐powered field (hpf) was examined using the Image‐Pro Plus 6.0 software. The data were presented as the average results of 10 random hpf.

### 2.4 Correlation analysis

The correlations among cGAS–STING pathway factors and the association between cGAS–STING pathway factor expression and clinical outcomes, including the uterine volume, and menstrual pain scores were analyzed with Spearman's rank correlation using the GraphPad Prism v6 software. Specifically, the uterine volume was determined by MRI scan. The menstrual pain score assessment was based on the visual analogue pain scale (VAS, scale: 0–10, 0 indicates no pain and 10 indicates the highest pain level) as described.^28^ The VAS scores were evaluated during the menstrual period and before the surgery of hysterectomy. The evaluating was performed every day, and the highest score among a menstrual period was chosen and determined as the VAS score for this particular menstrual period. If more than one menstrual cycle was evaluated for a given patient, the final score was calculated by the mean of all VAS scores.

### 2.5 Statistical analysis

The data analysis was performed using GraphPad Prism version 6 (GraphPad Software). The nonparametric Mann–Whitney *U* test was used to assess differences in parameters between the control and adenomyosis patient samples.

## 3 RESULTS

### 3.1 Demographic data

**Figure 1:**
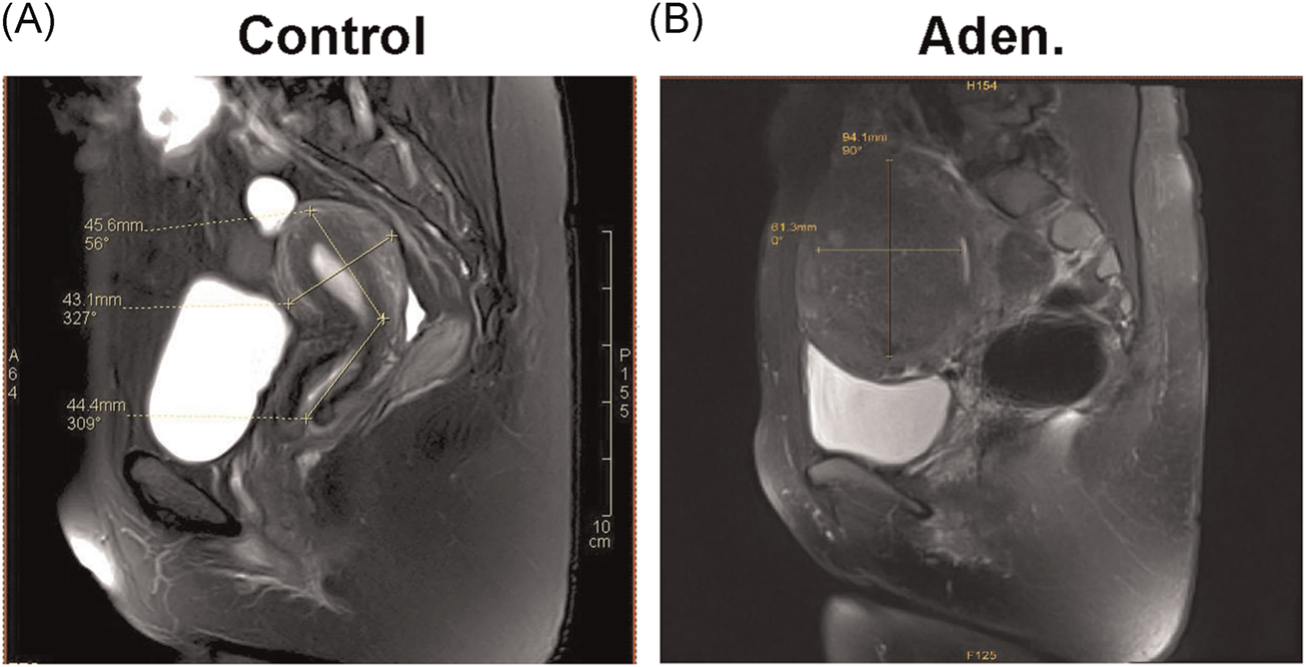
Representative pelvic MRI from the control and adenomyosis patients. (A) In a control patient (control), the MRI picture shows the normal uterine, with dimensions of a 45.6‐mm length and 43.1‐mm width crossing the whole uterus. (B) In an adenomyosis patient (Aden.), the MRI scan shows typical focal adenomyosis, with a 40.6‐mm length and 56.9‐mm width of pathological foci located in the uterine junctional zone (indicated with a red star). MRI, magnetic resonance imaging

The adenomyosis patients included in this study were initially diagnosed by MRI. Then the diagnosis of the patients was confirmed by histological staining. The demographic data from the adenomyosis and control patients were summarized in Table 1. The age, number of pregnancies, menstrual cycle length, and menstrual period length were similar between the adenomyosis and control patients, with no significant differences found between the two groups. However, the dysmenorrhea pain scores of 3.55 ± 3.35 in the adenomyosis patients were significantly higher than those of the control patients (*p* < .001). The uterine volume measured by MRI of 369.37 ± 155.70 cm^3^ for the adenomyosis patients was also significantly higher than the 83.86 ± 44.29 cm^3^ measured for the control patients (*p* < .001) (Table 2 and Figure 1).

**Table 2:** Characterization of the adenomyosis and control patients

Characteristic	Adenomyosis patients (*n* = 20)	Control patients (*n* = 10)	*p* value
Age (years)	45.55 ± 3.73	44.60 ± 3.06	.493
Number of pregnancies	3.70 ± 1.53	4.60 ± 2.17	.197
Menstrual cycle length (days)	27.85 ± 2.52	28.90 ± 1.85	.209
Menstrual period length (days)	5.85 ± 2.81	5.40 ± 0.84	.516
Dysmenorrhea (VAS pain scores)	3.55 ± 3.35	0 ± 0	<.001
Uterine volume (cm^3^, by MRI)	369.37 ± 155.70	83.86 ± 44.29	<.001

Abbreviations: MRI, magnetic resonance imaging; VAS, visual analogue pain scale.

*Note*: The data are presented as the mean ± *SD*, and the statistical analysis was performed with the Mann–Whitney *U* test except for the percentage of women with the symptom of heavy menstrual bleeding, which was analyzed with Fisher's exact test.

### 3.2 The mRNA levels of key cGAS–STING pathway molecules in adenomyosis patients

**Figure 2:**
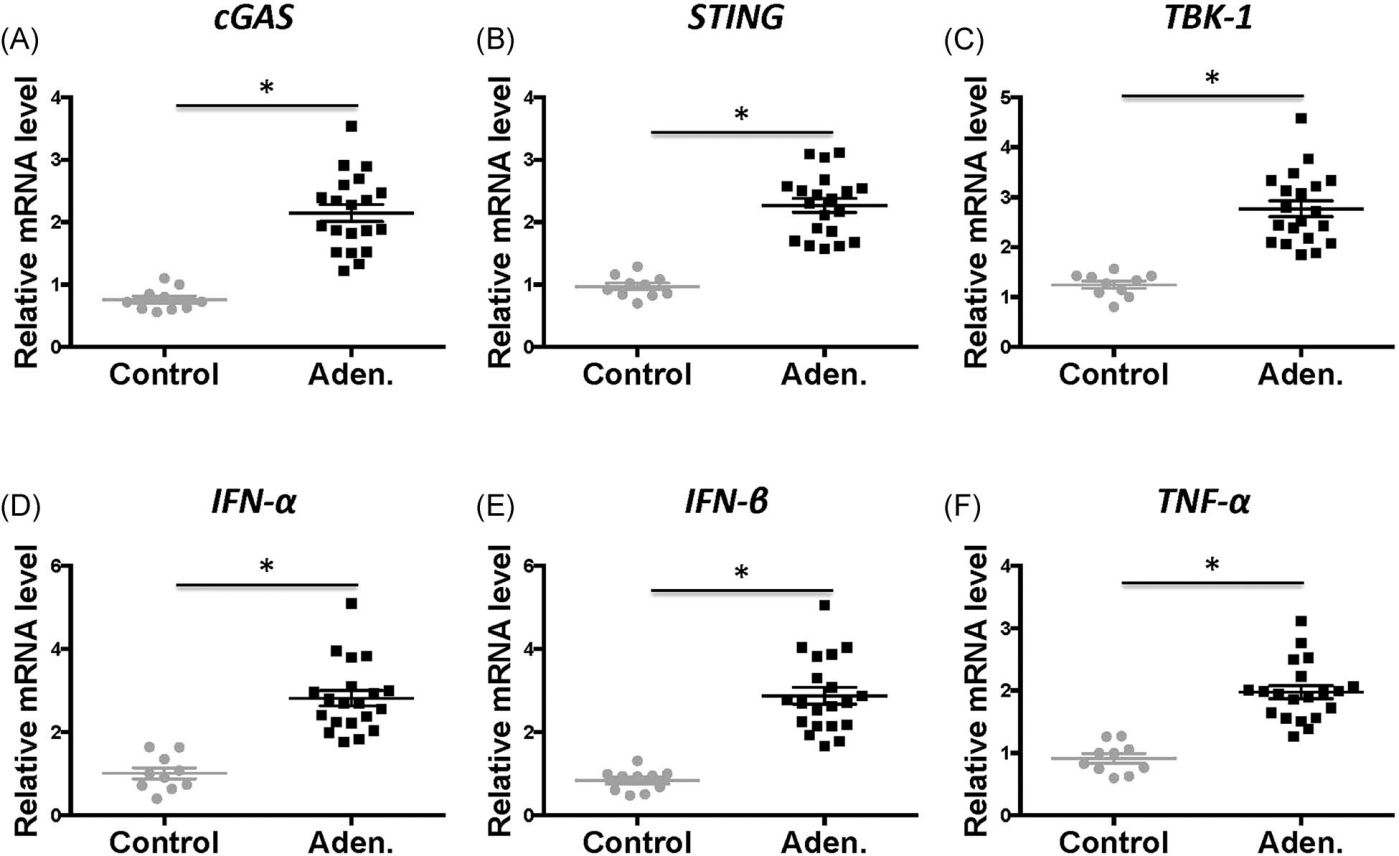
mRNA expression of cGAS–STING signals in the uterine tissue. Total mRNA was extracted from uterine tissues from the control and adenomyosis (Aden.) patients. Then, the mRNA was reverse‐transcription into cDNA and subjected to real‐time PCR to determine the expression of cGAS–STING signal pathway factors. The relative expression of (A) cGAS, (B) STING, (C) TBK‐1, (D) IFN‐α, (E) IFN‐β, and (F) TNF‐α was normalized to GAPDH and presented. *n* = 10 control patients, and *n* = 20 adenomyosis patients. The Mann–Whitney *U* test was performed to compare the control and aden. samples, *p < .05. cDNA, complementary DNA; GAPDH, glyceraldehyde 3‐phosphate dehydrogenase; IFN‐α, interferon‐α; mRNA, messenger RNA; PCR, polymerase chain reaction; TBK‐1, TANK‐binding kinase 1; TNF‐α, tumor necrosis factor‐α

Total RNA from the uterine tissue were extracted and qRT‐PCR was performed to measure the gene expression of key cGAS–STING pathway factors. Interestingly, the expression of all tested genes, including cGAS, STING, TBK‐1, IFN‐α, IFN‐β, and TNF‐α, were significantly higher in the adenomyosis patients than that in the control patients (Figure 2, *p* < .05, aden. vs. control). The expression levels were increased by 2–3‐fold for all tested genes.

### 3.3 Immunohistochemical staining of key cGAS–STING pathway molecules in adenomyosis patients

**Figure 3:**
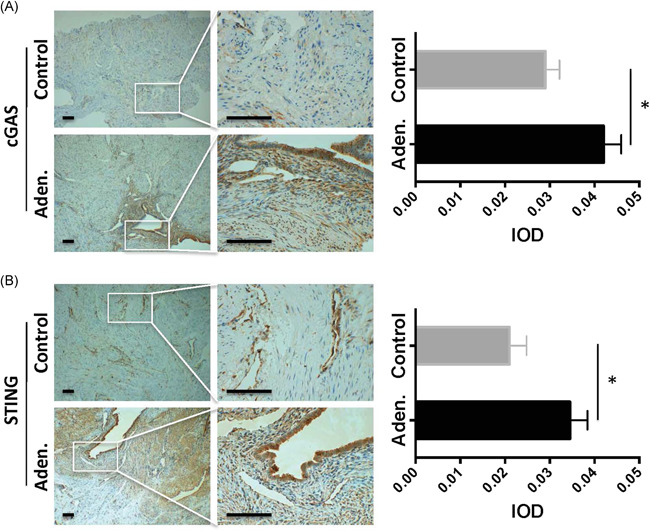
Local cGAS–STING protein expression in the uterine tissue. IHC staining of (A) cGAS and (B) STING in the uterine tissue from the control and adenomyosis (Aden.) patients. cGAS and STING were highly elevated in the adenomyosis patients but not in the control patients. The signals were strongly stained in the ectopic endometrial glands. The quantitative analysis confirmed that cGAS and STING staining was significantly higher in the adenomyosis patients than that in the control patients (A, B,  right panels, *p* < .05, aden. vs. control). Scale bar = 100 μm. The IOD per high‐powered field (hpf) was calculated for the IHC‐stained images, and the data were presented as the average result of 10 random high‐powered fields on the right side in the indicated panel. The Mann–Whitney *U* test was performed to compare the control and aden. samples, *p < .05. IHC, immunohistochemistry; IOD, integrated optical density

Next, the protein expression levels in the local tissues were evaluated by IHC staining. Our results showed that cGAS and STING were highly elevated in the adenomyosis patients but not in the control patients (Figure 3A,B, left panels). Especially, the signals were strongly stained in the ectopic endometrial glands. Furthermore, the quantitative analysis confirmed that cGAS and STING staining was significantly higher in the adenomyosis patients than that in the control patients (Figure 3A,B, right panels, *p* < .05, aden. vs. control).

**Figure 4:**
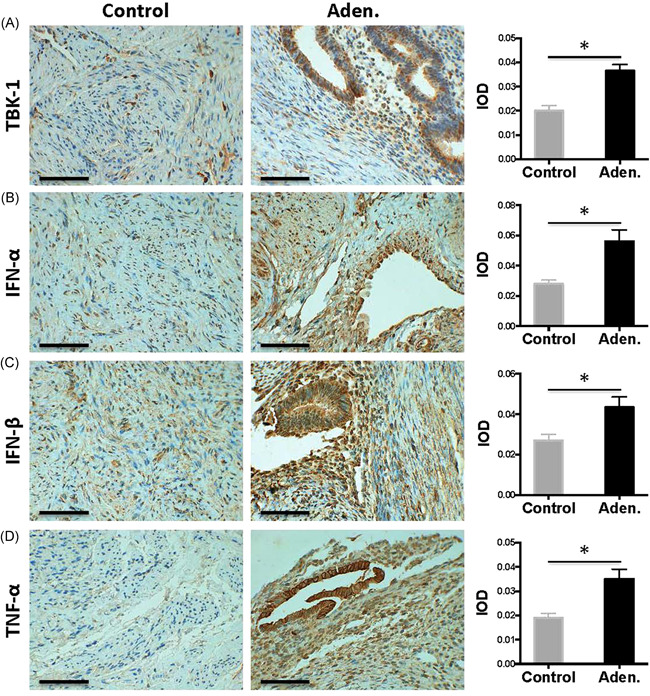
Local protein expression of cGAS–STING downstream factors in the uterine tissue. Representative IHC staining of (A) TBK‐1, (B) IFN‐α, (C) IFN‐β, and (D) TNF‐α in the uterine tissue is shown on the left. TBK‐1, IFN‐α, IFN‐β, and TNF‐α showed much stronger signals in the adenomyosis patients than that in the control patients based on the images and quantitative calculations (*p* < .05). Scale bar = 100 μm. IHC staining was quantified as the IOD per hpf and shown on the right side of the indicated panel. The data are presented as the average result of 10 random high‐powered fields. The Mann–Whitney *U* test was performed to compare the control and aden. samples, *p < .05. hpf, high‐powered field; IFN‐α, interferon‐α; IHC, immunohistochemistry; integrated optical density; TBK‐1, TANK‐binding kinase 1; TNF‐α, tumor necrosis factor‐α

In addition to cGAS and STING, the expression of cGAS–STING pathway downstream factors was also analyzed, including TBK‐1, IFN‐α, IFN‐β, and TNF‐α, in the local uterine tissue by IHC. All these factors showed much stronger signals in the adenomyosis patients than that in the control patients based on the images and quantitative calculations (*p* < .05) (Figure 4A–D). Furthermore, the staining pattern was similar to that of the cGAS–STING proteins as highly enriched in the ectopic endometrial glands.

### 3.4 Correlation analysis of cGAS–STING factors with clinical outcomes in adenomyosis patients

**Figure 5:**
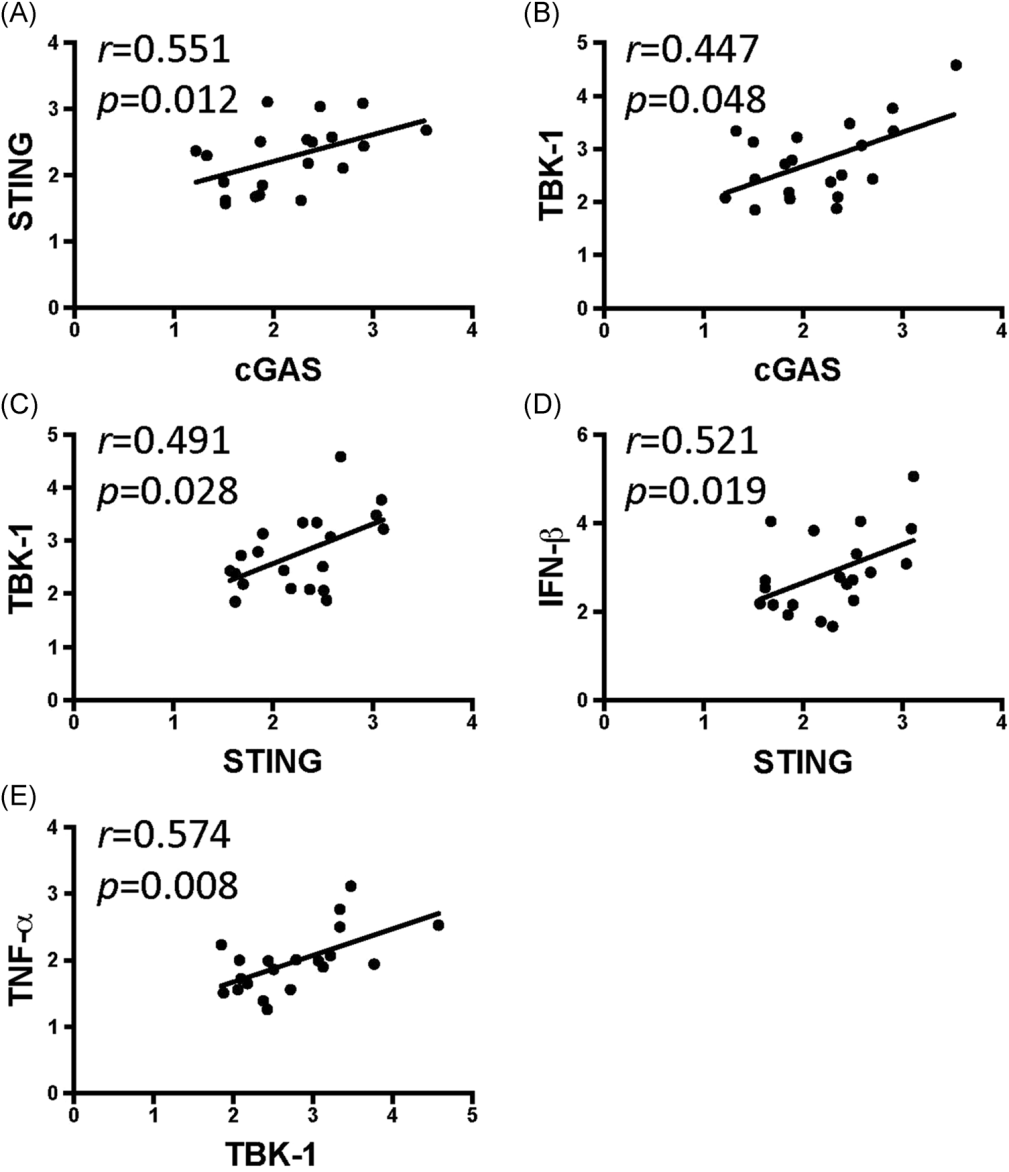
Correlation analysis between cGAS–STING signal pathway factors in adenomyosis patients. Correlation analyses between cGAS and (A) STING and (B) TBK‐1, between STING and (C) TBK‐1 and (D) IFN‐β, and (E) between TBK‐1 and TNF‐α were performed using Spearman's rank correlation. The coefficient (*r*) and *p* values (*p*) are presented. *p* < .05 indicates a significant correlation. IFN‐β, interferon‐β; TBK‐1, TANK‐binding kinase 1; TNF‐α, tumor necrosis factor‐α

**Figure 6:**
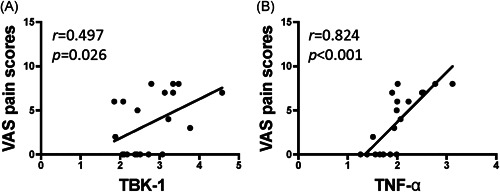
Correlation analyses between cGAS–STING signal pathway factors and dysmenorrhea in adenomyosis patients. Correlation analyses between cGAS–STING factors (A) TBK‐1 and (B) TNF‐α and dysmenorrhea (evaluated by the menstrual VAS pain scores) were performed using Spearman's rank correlation. The coefficient (*r*) and *p* values (*p*) are presented. Evaluation of the menstrual pain VAS pain scores is described in Section Materials and Methods, 2. *p* < .05 indicates a significant correlation. TBK‐1, TANK‐binding kinase 1; TNF‐α, tumor necrosis factor‐α; VAS, visual analogue pain scale

We evaluated the correlations among cGAS–STING pathway factors and found that cGAS was significantly correlated with the expression of STING (Figure 5A, *p* < .05) and TBK‐1 (Figure 5B, *p* < .05), while STING was significantly correlated with TBK‐1 (Figure 5C, *p* < .05), and IFN‐β (Figure 5D, *p* < .05). In addition, TBK‐1 was significantly correlated with the expression of TNF‐α (Figure 5E, *p* < .05). The correlations between cGAS–STING signal expression and the clinical outcomes were evaluated. The menstrual pain scores were significantly correlated with the expression of TBK‐1 (Figure 6A, *p* < .05) and TNF‐α (Figure 6B, *p* < .05).

## 4 DISCUSSION

Adenomyosis is a gynecological condition as presenting endometrial glands and the stroma within myometrium. The foci of adenomyiosis may be either diffuse or focal. Typical clinical manifestations are heavy menstrual bleeding, pelvic pain, and subfertility.^1^, ^4^, ^29^, ^30^ Transvaginal sonography^31^ and MRI^32^ are common examination tools for the initial clinical diagnosis, but histopathological examination is required to confirm the results.^30^ Although many hypotheses have been proposed for the pathogenesis of adenomyosis, the precise molecular mechanisms are still not well understood.^4^, ^30^ In this study, our data showed that the expression of key cGAS–STING pathway factors was significantly increased in the adenomyotic lesions tissue of adenomyosis patients compared to that of control patients without adenomyosis. This expression pattern seems to be not related to the type of adenomyosis. Both diffuse and focal adenomyosis showed increase of cGAS–STING signals. In addition, cGAS–STING pathway molecule expression was correlated with the clinical outcomes, particular dysmenorrhea. To our knowledge, this is the first study demonstrating that the cGAS–STING pathway was activated in adenomyosis patients and might play an important role in adenomyosis pathogenesis.

Typically, adenomyosis is regarded as a type of sex steroid hormone aberration disease.^4^ The uterine dysfunction may be due to local hyperestrogenism because increased estrogen receptor (ER) expression promotes the “spread” of adenomyosis into the myometrium.^4^ Indeed, suppressive hormone treatments, such as continuous use of oral contraceptive pills, high‐dose progestin, the levonorgestrel‐releasing intrauterine system, danazol and gonadotropin‐releasing hormone agonists, provide beneficial clinical outcomes of adenomyosis.^5^, ^30^ However, recent studies have supported the hypothesis that immune‐inflammatory responses are also involved and play critical roles in adenomyotic development. Several inflammatory cytokines showed abnormal expression in adenomyosis patients, including IL‐1β,^17^, ^33^ IL‐6,^34^, ^35^ IL‐8,^33^ IL‐10,^15^, ^16^ TNF,^16^ NF‐κB,^12^ MCP‐1,^14^ and RANTES,^36^ while multiple signal pathways, including TLR4^37^ and MAPK/ERK,^13^ were involved. In addition, immune cells, such as macrophages, natural killer cells and T helper cells, were reported to participate in adenomyotic disease development.^26^, ^38^, ^39^ In this study, we observed that cGAS and STING, as well as downstream key factors, were highly expressed in the local adenomyotic foci, indicating persistent activation of an acute inflammatory response and maintenance of an active inflammatory microenvironment in the adenomyotic foci. These effects may contribute to the progression and development of adenomyosis in vivo.

cGAS is activated through recognition of double‐strand DNA, which is a prominent anti‐inflammatory response in bacterial and viral infections. How is cGAS activated during adenomyosis? Several methods may be involved, for example, cGAS may recognize apoptotic and necrotic damaged cellular DNA released from mitochondria or damaged cells. In patients with adenomyotic foci, expression of apoptotic genes has been observed,^40^ which can cause intracellular cGAS–STING pathway activation. This topic should be investigated in future studies.

Typically, two major downstream pathways were identified after v signal activation. One is STING–TBK‐1–IRF3 axle, and the other one is STING–IKK–NF‐κB axle to induce type I IFN responses.^41^, ^42^ In this study, we found that STING and TBK‐1 expressions were positively correlated with cGAS expression. TBK‐1 and IFN‐β were positively correlated with STING, indicated that in our model, the STING–TBK‐1–IRF3 signal is the dominant pathway to produce type I IFN responses. To explore the association of clinical outcomes to cGAS–STING activation, we evaluated the cGAS–STING key pathway factors to the clinical outcomes including uterine volume, and menstrual pain scores. Even with limited amount of patients, we are able to identify that TBK‐1 and TNF‐α were correlated with dysmenorrhea, which was consistent with a previous study that NF‐κB DNA‐binding activity was correlated with the severity of dysmenorrhea in adenomyosis.^12^ However, other clinical outcomes, such as menstrual bleeding should also be evaluated in the future study, since heavily menstrual bleeding is also an important clinical symposium for adenomyosis patients.^2^, ^3^

In conclusion, our study reveals that the cGAS–STING pathway is activated in adenomyosis patients and its activation is subsequently correlated with clinical outcomes, which suggests that the cGAS–STING pathway may contribute to adenomyosis pathogenesis.

## CONFLICT OF INTERESTS

The authors declare that there are no conflict of interests.

## AUTHOR CONTRIBUTIONS

Xin Sun and Luying Wang conceived the study. Yun Lin and Mingzhu Ye performed the experiments. Yun Lin, Mingzhu Ye, Ke‐nan Yu, Doctor of Medicine (M.D.), and Xinliang Deng contributed to samples collections and data analysis. Yun Lin and Xin Sun wrote the manuscript. And Xin Sun and Min Xue supervised the work.
